# Predicting antibody affinity changes upon mutations by combining multiple predictors

**DOI:** 10.1038/s41598-020-76369-8

**Published:** 2020-11-11

**Authors:** Yoichi Kurumida, Yutaka Saito, Tomoshi Kameda

**Affiliations:** 1 Artificial Intelligence Research Center, National Institute of Advanced Industrial Science and Technology (AIST), 2-4-7 Aomi, Koto-ku, Tokyo 135-0064 Japan; 2 AIST-Waseda University Computational Bio Big-Data Open Innovation Laboratory (CBBD-OIL), 3-4-1 Okubo, Shinjuku-ku, Tokyo 169-8555 Japan; 3 Graduate School of Frontier Sciences, University of Tokyo, 5-1-5 Kashiwanoha, Kashiwa, Chiba 277-8561 Japan

**Keywords:** Protein analysis, Protein design

## Abstract

Antibodies are proteins working in our immune system with high affinity and specificity for target antigens, making them excellent tools for both biotherapeutic and bioengineering applications. The prediction of antibody affinity changes upon mutations (${{\Delta \Delta {\mathrm{G}}}}_{\mathrm{binding}}$) is important for antibody engineering. Numerous computational methods have been proposed based on different approaches including molecular mechanics and machine learning. However, the accuracy by each individual predictor is not enough for efficient antibody development. In this study, we develop a new prediction method by combining multiple predictors based on machine learning. Our method was tested on the SiPMAB database, evaluating the Pearson’s correlation coefficient between predicted and experimental ${{\Delta \Delta {\mathrm{G}}}}_{\mathrm{binding}}$. Our method achieved higher accuracy (R = 0.69) than previous molecular mechanics or machine-learning based methods (R = 0.59) and the previous method using the average of multiple predictors (R = 0.64). Feature importance analysis indicated that the improved accuracy was obtained by combining predictors with different importance, which have different protocols for calculating energies and for generating mutant and unbound state structures. This study demonstrates that machine learning is a powerful framework for combining different approaches to predict antibody affinity changes.

## Introduction

Antibodies are proteins working in our immune system that bind to target molecules named antigen such as proteins or chemical ligands with high affinity and specificity. Over the past two decades, antibodies have become popular as biotherapeutics^1^. Antibodies have important advantages over small-molecule drugs such as antibody dependent cellular cytotoxicity^2^ and complement dependent cytotoxicity activity^3^. In addition, antibody–drug conjugates can kill tumor cells with high efficiency^4,5^. Recently, a single chain fragment variable region of an antibody is used as a receptor for chimeric antigen receptor T-cell therapy^6,7^, highlighting the adaptability and efficacy of antibodies as biotherapeutics. Antibody engineering is used to improve the properties of antibodies such as affinity, specificity, solubility, and stability. In particular, improving affinity is important for increasing drug efficacy and decreasing the amount of antibody per dose, thereby reducing the drug price. The affinity of an antibody can be improved by introducing mutations in its amino acid sequence while in practice not many mutations increase affinity^8^. To date, improving affinity requires trial and error, making many mutants and measuring their affinities to identify mutants of interest.

The affinity of an antibody is evaluated by the binding free energy (${\mathrm{\Delta G}}_{\mathrm{binding}}$). ${\mathrm{\Delta G}}_{\mathrm{binding}}$ is calculated by the free energy of the bound state minus that of the unbound state. ${\mathrm{\Delta G}}_{\mathrm{binding}}$ is experimentally measured with surface plasmon resonance (SPR), isothermal titration calorimetry (ITC), or enzyme-linked immune-sorbent assay. Although SPR and ITC have high sensitivity, measuring many samples with SPR and ITC requires substantial time and cost. Therefore, it is important for antibody engineering to develop a method for predicting mutants with high affinity prior to experimental evaluation^9,10^.

A number of software tools have been developed for predicting binding affinity of complexes^11,12^, some of which are proposed for general protein complexes while others are dedicated specifically to antibody-antigen complexes^13,14^. These methods are largely divided into two approaches: molecular mechanics and machine learning. The molecular mechanics methods are based on the evaluation of energies calculated from protein structures^15,16^. Each method utilizes a different scoring function to calculate energies. The typical terms considered in a scoring function include hydrogen bonding, conformational energies, solvation energies, and entropic terms in addition to Coulombic and van der Waals interaction energies^17^. Normally, the molecular mechanics methods take as input the structure of a wild-type complex only, and mutant structures and structures in the unbound state are computationally generated (i.e. structure regeneration). Therefore, the performance of molecular mechanics methods depends on the choice of scoring functions and structure regeneration methods. Sulea et al.^17^ have presented a benchmark study to investigate the effect of scoring functions and structure regeneration methods on the prediction accuracy. As an approach different from molecular mechanics, the machine learning methods are proposed based on statistical models that predict affinity changes upon mutations using feature values calculated from protein complex structures^13,18^. The performance of machine learning methods is determined by the choice of statistical models and feature values.

Sulea et al.^17^ have also proposed a prediction method in their benchmark study. Their prediction method, termed consensus scoring, is defined as the average of predicted affinity changes calculated by multiple molecular mechanics methods (multiple predictors). In detail, the Z score is calculated for each of predictors for adjusting their difference in mean and standard deviation. Then, the consensus score is calculated as the average of the Z scores of predictors. The consensus scoring method has shown higher prediction accuracy than any of individual molecular mechanics methods (single predictors). However, the consensus scoring method does not consider the different importance of predictors since the method simply takes the average of the Z scores of predictors, assuming all features are equally important. In addition, the predictors used in the consensus scoring method have been selected empirically, thus the best combination of predictors for improving accuracy is unknown.

**Figure 1:**
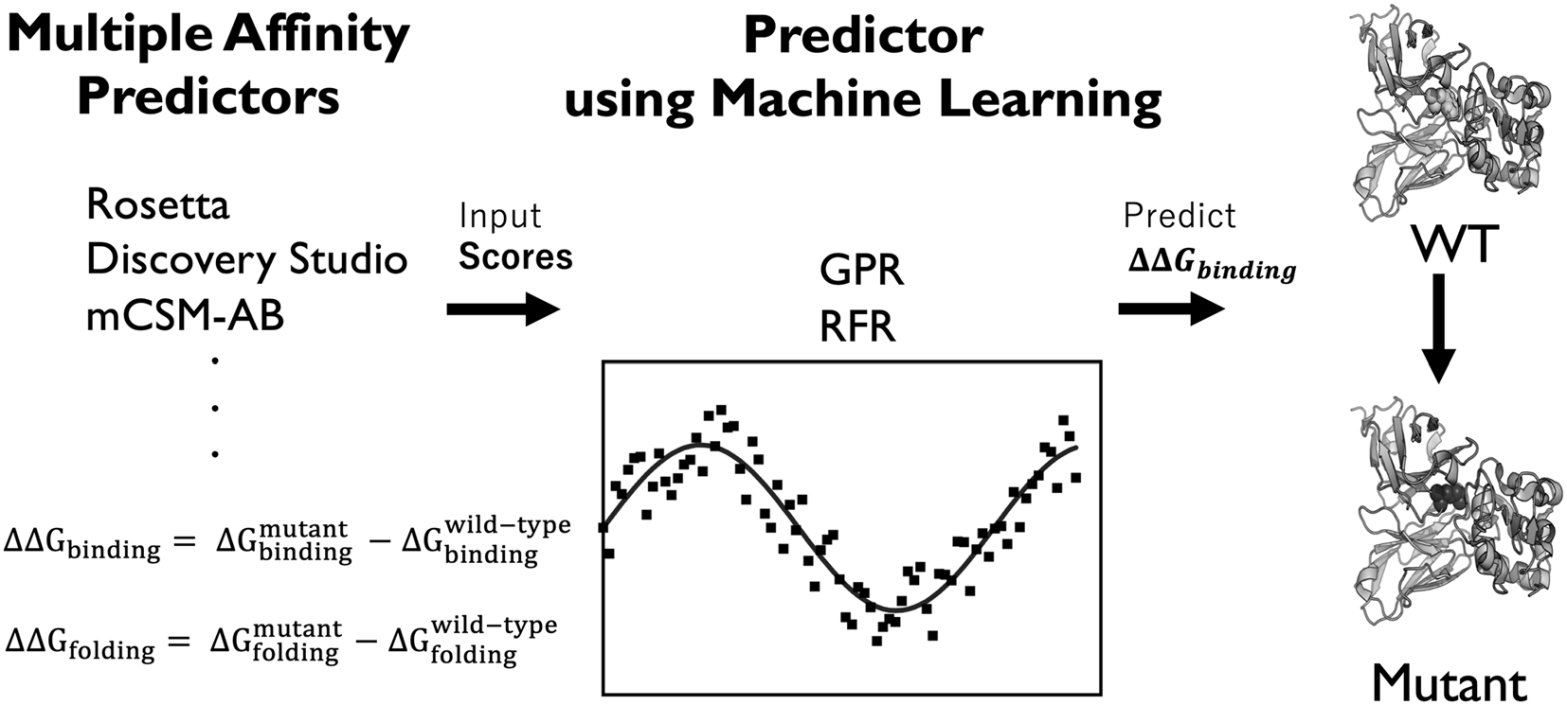
Overview of the proposed method. Our method uses predictions from multiple methods as feature values for machine learning models, and outputs ${{\Delta \Delta {\mathrm{G}}}}_{\mathrm{binding}}$ as the final prediction.

Here, we propose a new computational method for the prediction of antibody affinity changes upon mutations. Our method combines multiple predictors using machine learning. In contrast to the consensus scoring method based on the average of multiple predictors, the use of machine learning enables us to combine multiple predictors with different importance adjusted in model training. The machine learning model takes predictions from multiple methods as feature values (Fig. 1). These predictors include a variety of molecular mechanics predictors with various scoring functions and structure regeneration methods as well as a previous machine-learning-based predictor. In experiments on the SiPMAB database, our method achieves higher prediction accuracy than the best single predictor and the consensus scoring method. We present feature importance analysis to evaluate the contribution of each predictor in our method, showing that the improved accuracy is obtained by combining predictors using different scoring functions and structure regeneration methods. Moreover, we show that the number of combined predictors can be reduced according to the feature importance without compromising the accuracy.

## Results

### Prediction accuracy improved by combining multiple predictors

**Figure 2:**
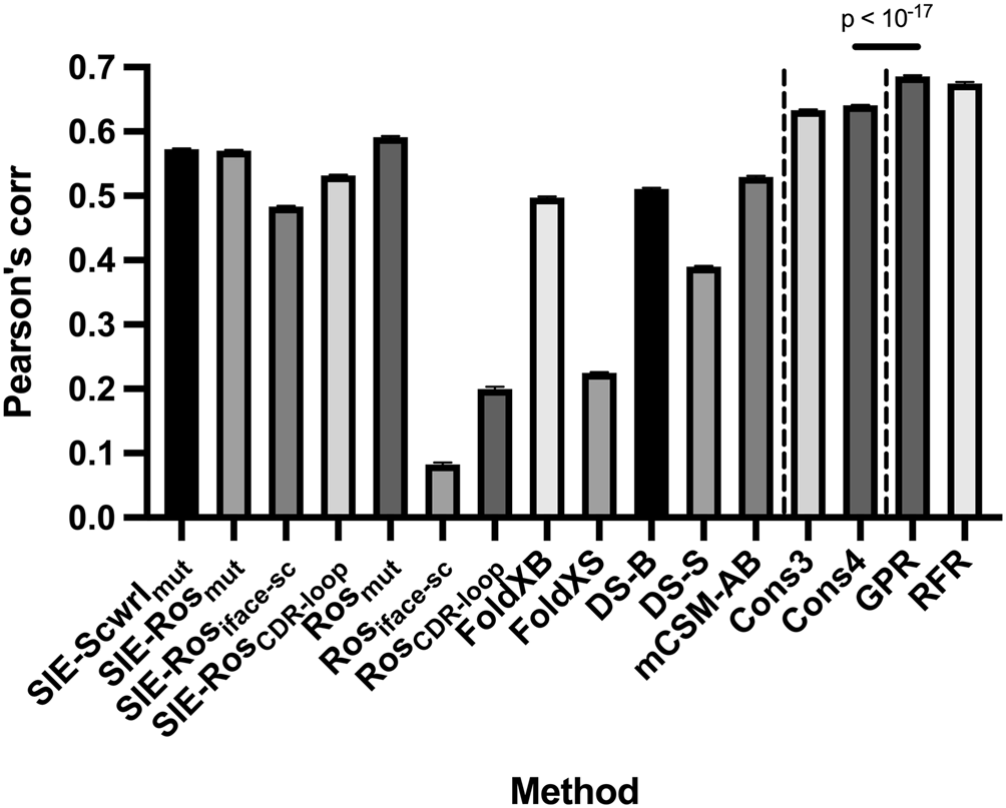
Comparison of different methods on the SiPMAB dataset. The bar graph shows the Pearson's correlation coefficient between predicted scores and experimental ${{\Delta \Delta {\mathrm{G}}}}_{\mathrm{binding}}$ in the SiPMAB dataset. Left: single predictors; Middle: Consensus scoring method; Right: the proposed method. The error bar represents the standard error of the mean (SEM) from 100 calculations using the different splits of subsets in cross validation. P-value was calculated using the Wilcoxon signed rank test.

We compared our method with the consensus scoring method based on the average of multiple predictors and the 12 kinds of single predictors used as feature values in our method (“Methods” section). As proposed in the previous study^17^, we used the consensus scoring method with 3 predictors (Cons3 with SIE-Scwrl_mut_, Ros_mut_ and FoldX-S) and that with 4 predictors (Cons4 with SIE-Scwrl_mut_, Ros_mut_, FoldX-S and FoldX-B). Figure 2 shows the Pearson’s correlation coefficient between predicted scores and experimental ${{\Delta \Delta {\mathrm{G}}}}_{\mathrm{binding}}$ on the SiPMAB dataset. Our method with GPR and RFR achieved R = 0.69 and R = 0.67, respectively, showing better accuracy than Cons3 (R = 0.63) and Cons4 (R = 0.64). These results demonstrate the effectiveness of machine learning for combining multiple predictors to improve the prediction accuracy.

The best single predictor was Ros_mut_ with R = 0.59. For each molecular mechanics software, R > 0.50 was achieved by using the best choice of scoring functions and structure regeneration methods: SIE-Scwrl_mut_, Ros_mut_, FoldX-B, and DS-B. The accuracy of FoldX-S and DS-S was lower than the other methods, which may be because these methods are based on the stability Eq. (3) rather than the binding free energy Eq. (1).

We compared the distribution of predicted scores for each method with experimental ${{\Delta \Delta {\mathrm{G}}}}_{\mathrm{binding}}$ (Supplementary Fig. S1). We found that there were outliers in the predictions of Ros_iface-sc_ and Ros_CDR-loop_. Notably, our method with GPR and RFR showed few outliers while it used these features. Such a robustness may be another merit of machine learning for combining multiple predictors.

### Analysis of feature importance

**Figure 3:**
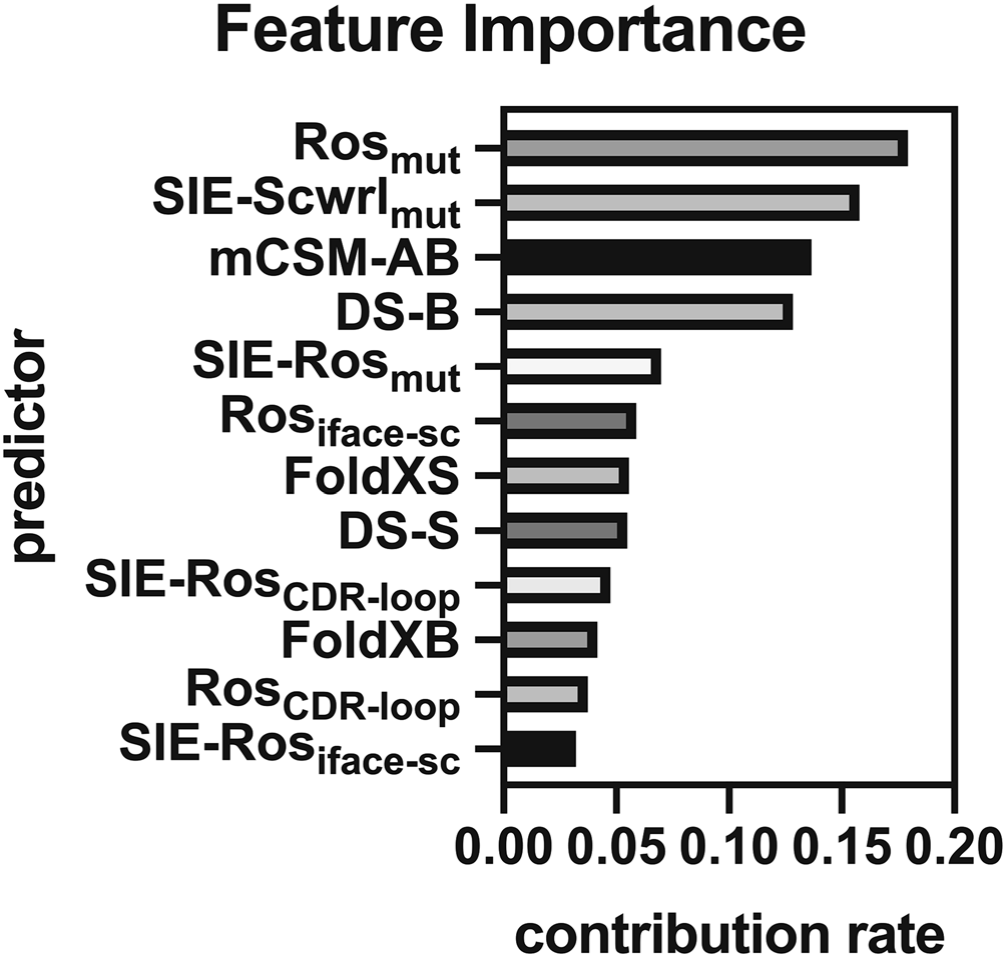
Feature importance analysis. The bar graph shows the feature importance of 12 predictors used in our method.

An advantage of machine learning is the ability to evaluate the importance of each feature in terms of its contribution to the prediction. We used the feature importance method based on Gini index^19^ implemented in scikit-learn package (Fig. 3). The most important feature was Ros_mut_, which also achieved the best accuracy among the single predictors (Fig. 2). Similarly, the feature with the second-highest accuracy, SIE-Scwrl_mut_, showed the second-highest feature importance whereas the tendency for the rank of accuracy and the rank of feature importance to become equal did not apply to the other features. The importance was above 0.1 for 4 features: Ros_mut_, SIE-Scwrl_mut_, mCSM-AB, and DS-B. Interestingly, those predictors were based on different prediction approaches (molecular mechanics or machine learning), and different scoring functions and structure regeneration methods for molecular mechanics. These results suggest that the improved accuracy of our method was obtained by combining predictors based on different principles.

### Correlation between predictors

**Figure 4:**
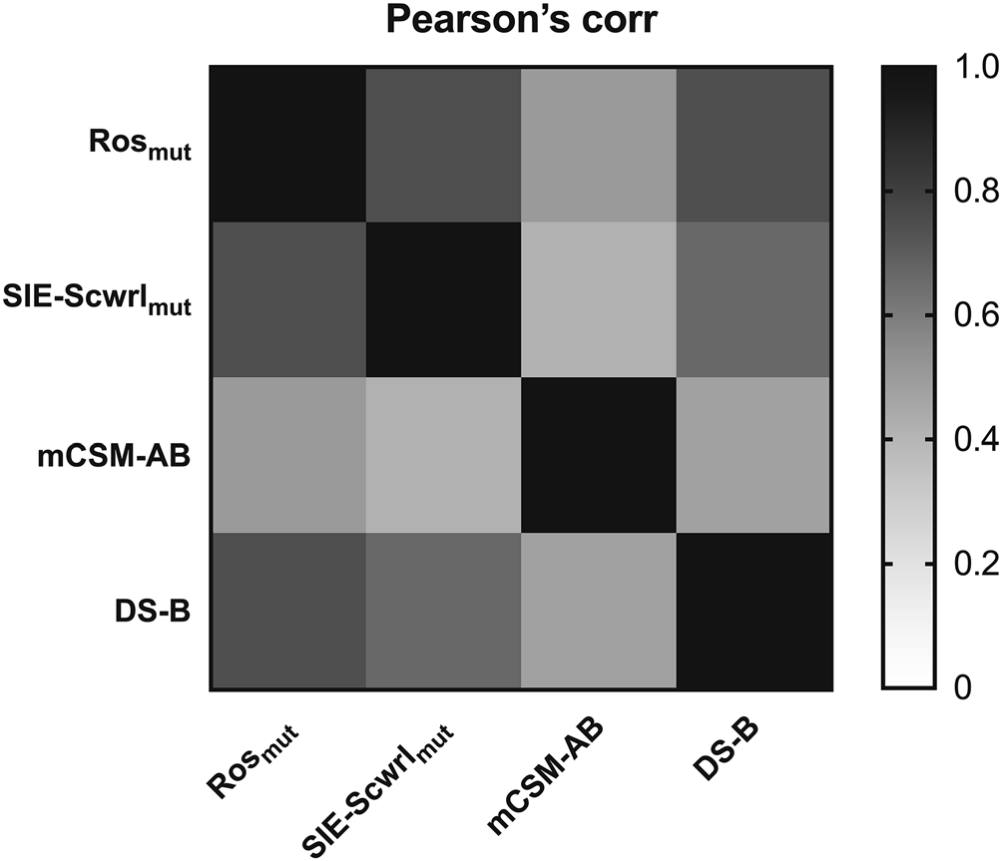
Correlation between the predictors. The heatmap shows the Pearson’s correlation coefficient between Ros_mut_, SIE-Scwrl_mut_, mCSM-AB and DS-B.

We also evaluated the Pearson’s correlation coefficient between different predictors (Fig. 4). Among the 4 predictors with high feature importance, the molecular mechanics predictors (Ros_mut_, SIE-Scwrl_mut_, and DS-B) were similar to each other (R > 0.66) with Ros_mut_ and DS-B showing the highest correlation. On the other hand, mCSM-AB based on machine learning was distinct from the other predictors (e.g. R = 0.50 between mCSM-AB and Ros_mut_). These results further support that combining predictors based on different principles may contribute to improving prediction accuracy.

### Reduced features

**Figure 5:**
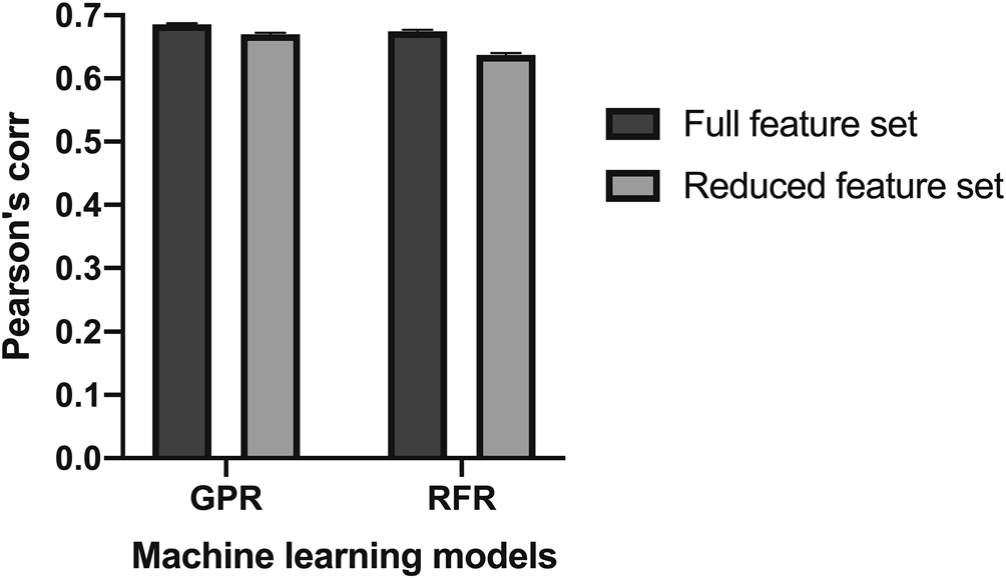
Comparison of accuracy using different feature sets. The bar graph shows the Pearson's correlation coefficient between predicted scores and experimental ${{\Delta \Delta {\mathrm{G}}}}_{\mathrm{binding}}$ in the SiPMAB dataset. Error bar represents the SEM from 100 calculations using different splits of subsets in cross validation.

Although our method uses 12 predictors as input, the number of predictors may be reduced, which is desirable for reducing the computational cost. Thus, we developed a prediction method combining only four predictors: Ros_mut_, SIE-Scwrl_mut_, mCSM-AB, and DS-B whose feature importance was higher than the others (Fig. 3). Our method using the reduced feature set achieved the accuracy comparable to that using the full feature set (Fig. 5). Using GPR as a machine learning model, the Pearson's correlation coefficient by our method was still higher than that of Cons4 (R = 0.67 compared with R = 0.64, P < 10^–15^; Wilcoxon signed-rank test). These results indicate that the number of features used for our method can be reduced without compromising prediction accuracy.

### Evaluation on independent data

**Figure 6:**
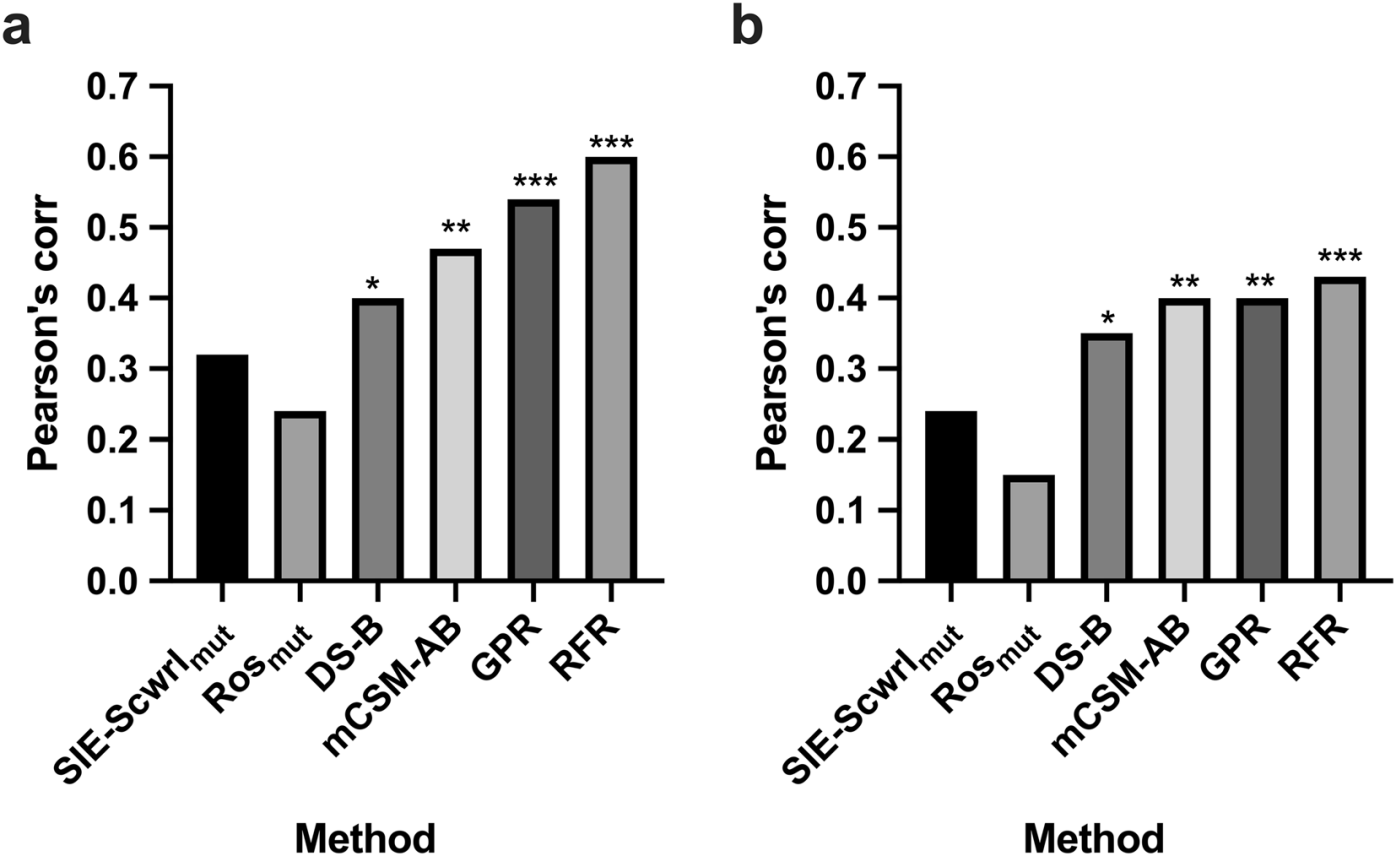
Comparison of different methods on independent data not included in SiPMAB database. The bar graph shows the Pearson's correlation coefficient between predicted scores and experimental ${{\Delta \Delta {\mathrm{G}}}}_{\mathrm{binding}}$. (**a**) bH1 data (n = 34). (**b**) bH1 data combined with 11K2 data (n = 46). *P < 0.05, **P < 0.01, ***P < 0.005.

In addition to the cross-validation-based evaluation on SiPMAB database, we performed a benchmark study on independent data not included in SiPMAB database (Methods). We compared our method using the reduced feature set with the 4 kinds of single predictors used as feature values in our method. Figure 6a shows the Pearson’s correlation coefficient between predicted scores and experimental ${{\Delta \Delta {\mathrm{G}}}}_{\mathrm{binding}}$ on 34 mutants of an antibody targeting vascular endothelial growth factor (VEGF), called bH1, from AB-Bind database^20,21^. Our method with GPR and RFR achieved R = 0.54 and R = 0.60, respectively, showing better accuracy than the best single predictor mCSM-AB with R = 0.47. We increased the data size by combining the bH1 data with additional independent data of 12 mutants of an antibody targeting monocyte chemo-attractant protein-1(MCP-1), called 11K2, from Kiyoshi et al.^22^, and also confirmed that our method achieved better accuracy than the single predictors (Fig. 6b). These results demonstrate the effectiveness of machine learning for combining multiple predictors to improve prediction accuracy, not only for SiPMAB database but also for independent data.

## Discussion

Numerous ${{\Delta \Delta {\mathrm{G}}}}_{\mathrm{binding}}$ prediction methods have been developed with a variety of scoring functions and structure regeneration methods. However, due to the characteristics of each method, ${{\Delta \Delta {\mathrm{G}}}}_{\mathrm{binding}}$ prediction with high accuracy has been difficult. In this study, we demonstrated that the prediction accuracy can be improved by combining multiple predictors using machine learning. Our method with GPR achieved R = 0.69 on the SiPMAB database (Fig. 2), which was more accurate than the best single predictor (Ros_mut_, R = 0.59) and the consensus scoring method based on the average of multiple predictors (Cons4, R = 0.64). The feature importance analysis suggested that Ros_mut_, SIE-Scwrl_mut_, mCSM-AB, and DS-B were particularly important for the improved accuracy (Fig. 3). Our method using these 4 features kept the prediction accuracy comparable to that using the full feature set (Fig. 5). Moreover, our method using these 4 features achieved higher accuracy than single predictors in the benchmark study on the independent data not included in SiPMAB database (Fig. 6). In addition, the feature importance analysis suggested that ${{\Delta \Delta {\mathrm{G}}}}_{\mathrm{folding}}$ (DS-S and FoldX-S) was not so important for the improved accuracy (Fig. 3).

The Pearson’s correlation coefficient between predictors ranged from 0.5 to 0.8 (Fig. 4). This result indicates that each predictor has unique information derived from different prediction approaches (molecular mechanics or machine learning), and different scoring functions and structure regeneration methods for molecular mechanics. In particular, the Pearson’s correlation coefficient between mCSM-AB and the other predictors based on molecular mechanics was lower than the other pairs. This result suggests that combining predictors based on molecular mechanics and machine learning is important for accuracy.

We note that our method has the limitations summarized below. First, although our method achieved higher accuracy than single predictors and the previous method using the average of multiple predictors, our methods requires a relatively high computational cost. Second, although our method achieved higher accuracy, it requires training data. On the other hand, the consensus scoring method and single predictors do not require training data. Third, our method, like other existing methods, requires the three-dimensional structure of the antigen–antibody complex. However, antibody-antigen complexes are easier to crystalize than monomers because the complexes are normally stable^23^. In addition, complex structures can be predicted using homology modeling^24,25^ and docking simulation^26^. In this study, we focused on affinity changes upon single point mutations as in previous studies. Nonetheless, our method can be easily extended to multiple point mutations by using scores of multiple point mutants as feature values.

In conclusion, our method performs the best for predicting affinity changes upon mutations of antibody-antigen complexes (${{\Delta \Delta {\mathrm{G}}}}_{\mathrm{binding}}$). The method is more accurate than the single predictors and the consensus scoring method using the average of multiple predictors. The improved accuracy is obtained by combining multiple predictors with different importance using machine learning. Our method can contribute to the design of antibodies for therapeutics and diagnostics by improving speed and reducing the associated costs.

## Methods

### Overview of the proposed method

The idea of our method is to combine multiple predictors for antibody affinity changes using machine learning (Fig. 1). The machine learning model takes as input predictions from multiple methods as feature values, and outputs the ${{\Delta \Delta {\mathrm{G}}}}_{\mathrm{binding}}$ as the final prediction. These predictors (feature values) included those based on molecular mechanics with different scoring functions and structure regeneration methods (Table S1). In addition, we also employed a previous machine-learning-based predictor as a feature value in our method (Table S1). We used two different machine learning models and compared their performance: gaussian process regression (GPR)^27^ and random forest regressor (RFR)^28^. GPR and RFR are one of the most popular machine learning models, which have been used for study such as antibody engineering field^29,30^. As an advantage of the use of machine learning, our method can evaluate the importance of each feature in terms of its contribution to the prediction. Specifically, we evaluated the feature importance based on the Gini index in RFR^19^. Our method was implemented in Python using scikit-learn package^31^.

### Predictors based on molecular mechanics

${\mathrm{\Delta G}}_{\mathrm{binding}}$ of an antigen–antibody complex is calculated with Eq. (1). ${\mathrm{G}}_{\mathrm{Ag}+\mathrm{Ab}}$ is the Gibbs free energy of the antigen–antibody complex. ${\mathrm{G}}_{\mathrm{Ag}}$ and ${\mathrm{G}}_{\mathrm{Ab}}$ are the Gibbs free energies of the unbound state of the antigen and the antibody, respectively.

1 $${\mathrm{\Delta G}}_{\mathrm{binding}}= {\mathrm{G}}_{\mathrm{Ag}+\mathrm{Ab}}-({\mathrm{G}}_{\mathrm{Ag}}+ {\mathrm{G}}_{\mathrm{Ab}})$$

The change in the binding energy after mutagenesis (${{\Delta \Delta {\mathrm{G}}}}_{\mathrm{binding}}$) is calculated with Eq. (2). ${\mathrm{\Delta G}}_{\mathrm{binding }}^{\mathrm{mutant}}$ and ${\mathrm{\Delta G}}_{\mathrm{binding }}^{\mathrm{wild}-\mathrm{type}}$ are the ${\mathrm{\Delta G}}_{\mathrm{binding}}$ of the mutant and the wild-type complexes, respectively.

2 $${{\Delta \Delta {\mathrm{G}}}}_{\mathrm{binding}}= {\mathrm{\Delta G}}_{\mathrm{binding }}^{\mathrm{mutant}}-{\mathrm{\Delta G}}_{\mathrm{binding }}^{\mathrm{wild}-\mathrm{type}}$$

The stability of an antigen–antibody complex is also calculated because it is related to binding free energy^17^. The stability (${\mathrm{\Delta G}}_{\mathrm{folding}}$) of an antigen–antibody complex is calculated with Eq. (3).

3 $${\mathrm{\Delta G}}_{\mathrm{folding}}= {\mathrm{G}}_{\mathrm{fold}}-{\mathrm{G}}_{\mathrm{unfold}}$$

${\mathrm{G}}_{\mathrm{fold}}$ and ${\mathrm{G}}_{\mathrm{unfold}}$ are the Gibbs free energies of the folded state and the unfolded state, respectively. The change in the structure stability after mutagenesis (${{\Delta \Delta {\mathrm{G}}}}_{\mathrm{folding}}$) is calculated with Eq. (4) ${\mathrm{\Delta G}}_{\mathrm{folding }}^{\mathrm{mutant}}$ and ${\mathrm{\Delta G}}_{\mathrm{folding }}^{\mathrm{wild}-\mathrm{type}}$ are the ${\mathrm{\Delta G}}_{\mathrm{folding}}$ of the mutant and the wild-type complexes, respectively.

4 $${{\Delta \Delta {\mathrm{G}}}}_{\mathrm{folding}}= {\mathrm{\Delta G}}_{\mathrm{folding }}^{\mathrm{mutant}}-{\mathrm{\Delta G}}_{\mathrm{folding }}^{\mathrm{wild}-\mathrm{type}}$$

In this study, we used 11 molecular mechanics predictors as feature values in our method. Among them, 9 predictors have been evaluated in the previous benchmark study^17^, while 2 predictors were newly employed in this study. Each predictor was different in the choice of a scoring function and a structure regeneration method, in addition to whether it used the binding energy Eq. (1) or the stability Eq. (3). The scoring functions included SIE^32^, Talaris2013^15^, Talaris-interface^33^, CHARMm Polar H^16^ and FOLDEF^34^. For regenerating mutant structures from the wild-type complex structure, only the side chain at the mutated site was repacked with the other residues fixed, or the side chains around the mutated site were also repacked (see the details below). Structures in unbound state were refined by separating the antibody and the antigen as rigid bodies, or by refining their structures after the separation. Below, for clarity, we divide the 11 predictors into 4 groups: Discovery Studio (2 predictors), FoldX (2 predictors), Rosetta (6 predictors), SIE-Scwrl_mut_ (1 predictor).

### Parent structure preparation

Predictors based on molecular mechanics require a parent structure that is prepared from an experimental structure for computational analyses. In this study, we used the parent structures provided by SiPMAB database for antibodies included in SiPMAB database. For other antibodies not included in SiPMAB database, we prepared the parent structures using the same procedure as SiPMAB database according to Sulea et al.^17^ Briefly, the starting structure was retrieved from the protein data bank (PDB ID: 3BDY for the anti-VEGF antibody; 2BDN for the anti-MCP-1-antibody), and we removed non-protein compounds including waters and ions, and deleted non-variable domains in the antibody. Protons were added with neutral pH condition. The structure was energy-minimized using Amber force field^35,36^.

### Discovery studio

Discovery Studio^37^ is biomolecular simulation software where CHARMm Polar H force field^16^ is used as a scoring function. Two types of protocols were used by Discovery Studio 2018: DS-B and DS-S. ${{\Delta \Delta {\mathrm{G}}}}_{\mathrm{binding}}$ (DS-B) was calculated by the “Calculate Mutation Energy (Binding)” protocol, and ${{\Delta \Delta {\mathrm{G}}}}_{\mathrm{folding}}$(DS-S) was calculated by the “Calculate Mutation Energy (Stability)” protocol. The structure of the mutant was refined with repacking and energy minimization of the side chain at the mutated site. The structures in unbound state were refined by rigid separation. All runs were performed with default parameters.

### FoldX, SIE-Scwrl_mut_, and Rosetta

Predictions for SiPMAB by FoldX^34^ (FoldX-B and FoldX-S), SIE-Scwrl_mut_^17^, and Rosetta^38^ (SIE-Ros_mut_, SIE-Ros_iface-sc_, SIE-Ros_CDR-loop_ , Ros_mut_, Ros_iface-sc_, and Ros_CDR-loop_) were obtained from the previous benchmark study^17^. Predictions for the complex of anti-VEGF antibody and VEGF were calculated according to the previous benchmark study^17^. The descriptions of these methods were shown in Table S1. Briefly, FoldX is protein free energy calculation software using FOLDEF as a scoring function. Two types of protocols were used by FoldX: FoldX-B and FoldX-S using ${{\Delta \Delta {\mathrm{G}}}}_{\mathrm{binding}}$ and ${{\Delta \Delta {\mathrm{G}}}}_{{{\text{folding}}}}$, respectively. SIE-Scwrl_mut_ uses 2 software and a scoring function: SCWRL is software for regenerating protein structures based on empirical side chain rotamers. Amber is software for molecular dynamics simulation and SIE is a scoring function^32,39^. In this protocol, mutant structures after refined by SCWRL with repacking and energy minimization of mutated side chains were further energy-minimized around mutated sites using Amber, and then ${{\Delta \Delta {\mathrm{G}}}}_{\mathrm{binding}}$ was calculated using SIE. Rosetta suite^40^ is a protein design and structure prediction tool based on Monte Carlo simulation. It is capable of predicting scores and generating a mutant structure with backrub sampling of the backbone and repacking of side chains. Six types of protocols were used by Rosetta: SIE-Ros_mut_, SIE-Ros_iface-sc_, SIE-Ros_CDR-loop_, Ros_mut_, Ros_iface-sc_, and Ros_CDR-loop_. Rosetta employed 3 scoring functions: Talaris2013^15^ (Ros_iface-sc_ and Ros_CDR-loop_), Talaris-interface^33^ (Ros_mut_), and SIE^32^ (SIE-Ros_mut_, SIE-Ros_iface-sc_, and SIE-Ros_CDR-loop_).

### Machine-learning-based predictor (mCSM-AB)

In addition to molecular mechanics predictors described above, we also used a previous machine-learning-based predictor, mCSM-AB^13^, as a feature value in our method. mCSM-AB is a machine learning model that predicts antibody affinity changes using the graph-based signatures of protein structures. In the previous study, the model has been trained using experimental ${{\Delta \Delta {\mathrm{G}}}}_{\mathrm{binding}}$ from the AB-Bind database^20^. To use mCSM-AB in our method, we took care to prevent the leakage of training data into the performance evaluation (see the “Performance evaluation” section for details).

### Datasets

To assess prediction accuracy, a dataset from the SiPMAB database^17^ was used. This dataset is comprised of 212 single point mutant antibodies in their CDRs, across 7 different antibody-antigen complexes. The wild-type structures of the antibody-antigen complexes are available, which are solved by high resolution X-ray crystallography. The majority of experimental binding free energies are measured by SPR and ITC. The ${{\Delta \Delta {\mathrm{G}}}}_{\mathrm{binding}}$ values range between − 0.65 and 7.32 kcal/mol. When the multiple ${{\Delta \Delta {\mathrm{G}}}}_{\mathrm{binding}}$ measurements are recorded for the same mutant, which originates from different publications, we selected one ${{\Delta \Delta {\mathrm{G}}}}_{\mathrm{binding}}$ value as previously described^17^. Briefly, the ${{\Delta \Delta {\mathrm{G}}}}_{\mathrm{binding}}$ value was selected considering the reliability of assay methods, and the scale of the assay in the original publication. Although AB-Bind^20^ and SKEMPI^41^ are another database for affinity changes upon mutations, we used the SiPMAB database since it collects mutants on antibodies excluding mutants on antigens, and thus is more suitable for the purpose of our study.

For evaluation on independent data, 34 mutants of the complex of anti-VEGF antibody, called bH1, and VEGF were collected from the AB-Bind. These mutants are not included in SiPMAB database, and have mutations in the antibody side. We also used the data of 12 mutants of the complex of anti-MCP-1 antibody, called 11K2, and MCP-1 from Kiyoshi et al.^22^.

### Performance evaluation

We evaluated the Pearson's correlation coefficient between predicted and experimental ${{\Delta \Delta {\mathrm{G}}}}_{\mathrm{binding}}$ as a measure of prediction accuracy. To compare the performance of our method with previous methods, we conducted the following procedures to ensure a fair comparison avoiding potential overfitting.

For RFR, we used fourfold nested cross-validation for optimizing a hyperparameter in our method (the number of trees). In the outer loop of the nested cross-validation procedure, the dataset was split into 4 subsets where each subset was used as an independent test dataset named Test and the remaining data were used for the inner loop. In the inner loop of the nested cross-validation procedure, the dataset was split into 4 subsets where each subset was used as a validation dataset named Validation, and the remaining data were named Training. A model was trained using Training dataset with various hyperparameter values, and the performance was measured using Validation dataset. The performance of our method with the best hyperparameter value was evaluated using the Test dataset. The Pearson's correlation coefficient between predicted and experimental ${{\Delta \Delta {\mathrm{G}}}}_{\mathrm{binding}}$ was calculated for each Test dataset, and the average value was used as a final evaluation measure. For GPR, we used the radial basis function (RBF) kernel with a constant kernel and a white kernel. The hyperparameters of GPR can be optimized during model training without looking at validation or test datasets by maximizing log-marginal-likelihood as implemented in scikit-learn package. Thus, we conducted a fourfold cross-validation rather than a fourfold nested cross-validation.

One of the predictors used in our method, mCSM-AB, was itself based on machine learning. Therefore, we took care to ensure that the data used for training mCSM-AB were always separated from the training data of our method. The mCSM-AB implemented in the public web server (https://biosig.unimelb.edu.au/mcsm_ab/prediction) has been trained using the AB-Bind database. Thus, we checked the overlap of data between the AB-Bind and SiPMAB databases. For each mutant in the SiPMAB database, when the mutant did not exist in the AB-Bind, we simply used the mCSM-AB web server for calculating the feature value in our method. When the mutant existed in the AB-Bind, we used the mCSM-AB predictions in the "Predictions on cross validation" provided by the developers of mCSM-AB (https://biosig.unimelb.edu.au/mcsm_ab/data) rather than the web server. These mCSM-AB predictions were obtained from the tenfold cross-validation where the mutant was separated from the training data.

In addition to cross-validation-based evaluation above, we performed a benchmark study where our method was trained on SiPMAB database, and evaluated on independent data not included in SiPMAB database. For this purpose, we used the bH1 data of the anti-VEGF antibody and the 11K2 data from Kiyoshi et al.^22^.

## Supplementary information

Supplementary Table S1.

Supplementary Figure S1.

Supplementary Information 1.

## Data Availability

Our method was implemented in Python using scikit-learn package. The codes and datasets for reproducing the results in this study are available at the authors' GitHub website: https://github.com/ykurumida/ab-predictor.
