# Supplementary figures and images for: Predicting antibody affinity changes upon mutations by combining multiple predictors
Source: Sci Rep. 2020 Nov 11;10:19533. doi: 10.1038/s41598-020-76369-8 (PMC7658247; doi:10.1038/s41598-020-76369-8)

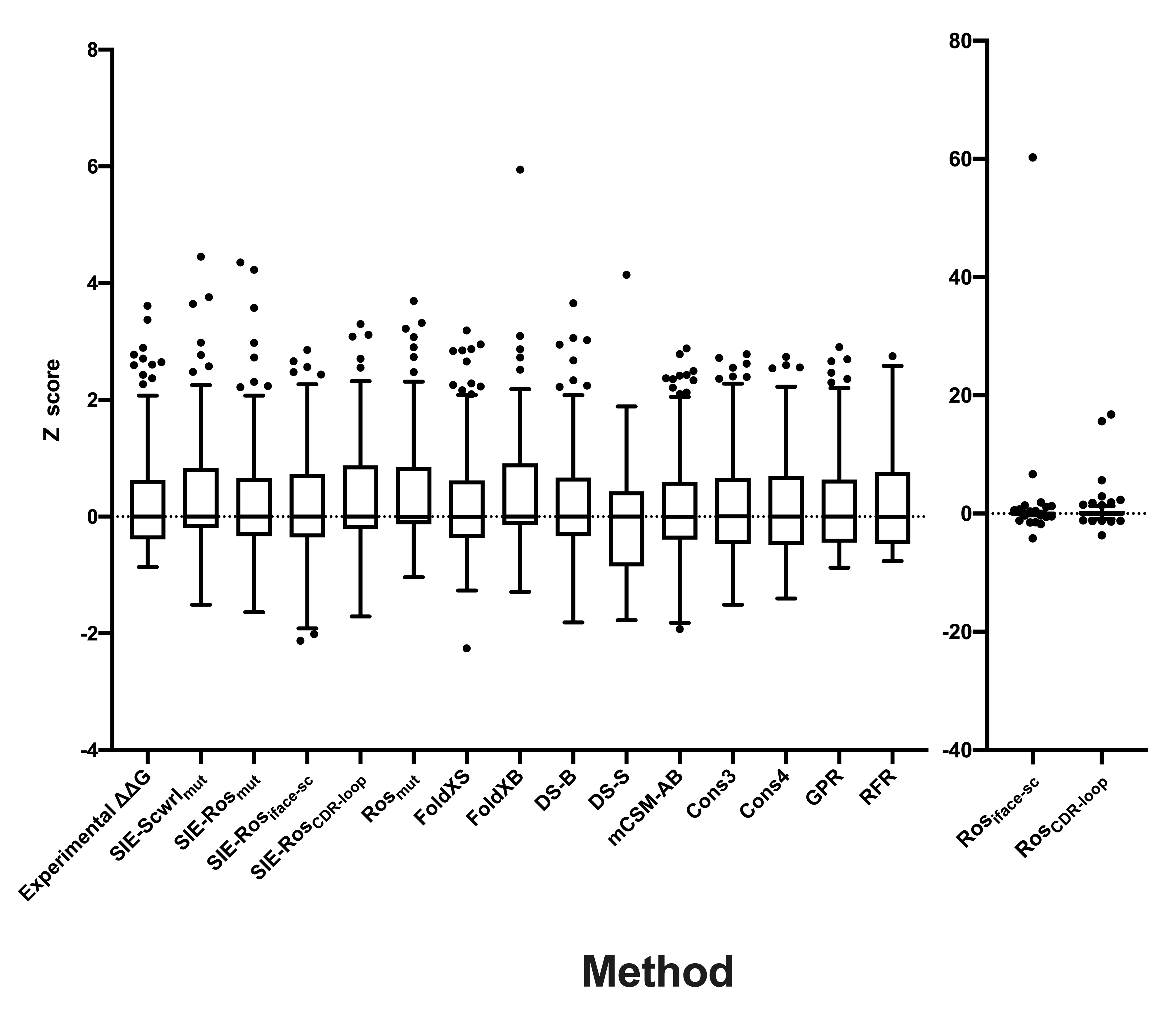

Supplement: Supplementary file 2 — Supplementary Figure S1. [file 41598_2020_76369_MOESM2_ESM.tiff]
