# Supplementary material for: Predicting antibody affinity changes upon mutations by combining multiple predictors
Source: Sci Rep. 2020 Nov 11;10:19533. doi: 10.1038/s41598-020-76369-8 (PMC7658247; doi:10.1038/s41598-020-76369-8)
Supplement: Supplementary file 3 — Supplementary Information 1. [file 41598_2020_76369_MOESM3_ESM.docx]

**Supplementary Information**

**Title**

Predicting Antibody Affinity Changes upon Mutations by Combining Multiple Predictors

**Authors**

Yoichi Kurumida^1^, Yutaka Saito^1,2,3^, Tomoshi Kameda^1*^

1 Artificial Intelligence Research Center, National Institute of Advanced Industrial Science and Technology (AIST), 2-4-7 Aomi, Koto-ku, Tokyo 135-0064, Japan.

2 AIST-Waseda University Computational Bio Big-Data Open Innovation Laboratory (CBBD-OIL), 3-4-1 Okubo, Shinjuku-ku, Tokyo 169-8555, Japan.

3 Graduate School of Frontier Sciences, University of Tokyo, 5-1-5 Kashiwanoha, Kashiwa, Chiba 277-8561, Japan.

**Contact:** [kameda-tomoshi@aist.go.jp](mailto:kameda-tomoshi@aist.go.jp)

**Legends for Supplementary Data**

**Supplementary Figure S1. Distribution of the predictions.** The box-and-whisker plot shows the distribution of the predictions of each method. For comparison, the distribution of experimental ${\Delta\Delta G}_{\mathrm{binding}}$ is also shown. To adjust different value scales of predictors, we showed the distributions of Z scores rather than raw values. Outliers were defined as data points whose Z scores are less than Q1-1.5*IQR or larger than Q3+1.5*IQR, where Q1 and Q3 are 25% and 75% quantiles, respectively, and IQR=Q3-Q1. As Ros_iface-sc_ and RosC_DR-loop_ had strong outliers, they were plotted separately.

**Supplementary Table S1.** Summary of the predictors. The table is available as a separate Excel file.
